# 17157 Racial/ethnic disparities in antibiotic-resistant infections: Knowledge gaps and opportunities for educational interventions

**DOI:** 10.1017/cts.2021.601

**Published:** 2021-03-30

**Authors:** Maya L. Nadimpalli, Courtney W. Chan, Shira Doron, Berri Jacque, Carol Bascom-Slack

**Affiliations:** 1 Tufts University; 2 Tufts University, School of Medicine, Tufts Medical Center; 3 Tufts University, School of Medicine

## Abstract

ABSTRACT IMPACT: By identifying clear gaps in our knowledge of racial and ethnic disparities in antibiotic-resistant infections, this research is informing the design of (a) community-based interventions and (b) patient-centered research studies that we are currently leading to address these disparities and improve human health. OBJECTIVES/GOALS: Antibiotic resistance (AR) is widely considered to be the next global pandemic. As with COVID-19, the potential for AR to disproportionately impact racial/ethnic minorities is a major concern. Our goal was to identify gaps in knowledge of AR disparities in order to inform the types of interventions that might be most appropriate to address this. METHODS/STUDY POPULATION: We reviewed the literature to examine evidence of racial/ethnic disparities in (a) infections with the most concerning drug-resistant bacteria in the United States, and (b) underlying social-economic or behavioral factors that could contribute to such infections. We searched PubMed and Google Scholar to identify studies published in English between August 1973 - August 2020. We used keywords that included: antibiotic resistance, antibiotic-resistant infections, antibiotic-seeking behavior, prescription/non-prescription antibiotic use, antibiotic education, or health literacy AND race, ethnicity, or socioeconomic status. We screened all abstracts to identify US-based studies that assessed (a) or (b) above. RESULTS/ANTICIPATED RESULTS: We identified 11 studies investigating racial/ethnic disparities for 5 of the 17 drug-resistant bacteria flagged in the CDC’s 2019 Antibiotic Resistance Threats Report. Black, Hispanic, and lower-income individuals were found to be at higher risk of some community-acquired antibiotic-resistant infections. We identified multiple factors that may contribute to disparities in AR-related morbidity and mortality, including reported differences in antibiotic use, higher likelihood of living in crowded/multigenerational homes, more frequent employment in potentially high exposure settings (e.g. slaughterhouses), lower health literacy, and more frequent underlying comorbidities, which increases risks for hospitalization and subsequent acquisition of drug-resistant infections. DISCUSSION/SIGNIFICANCE OF FINDINGS: Given the small number of studies on this topic, educational interventions that aim to raise awareness of this issue must target not only the public but also researchers. Community-based interventions that seek to address disparities in ‘antibiotic resistance literacy’ among minority and underserved groups could be particularly impactful.

