# 17157 Racial/ethnic disparities in antibiotic-resistant infections: Knowledge gaps and opportunities for educational interventions – ERRATUM

**DOI:** 10.1017/cts.2021.817

**Published:** 2021-07-26

**Authors:** Maya L. Nadimpalli, Courtney W. Chan, Shira Doron, Berri Jacque, Carol Bascom-Slack

The above abstract [1] published with the incorrect middle initial for Courtney W. Chan.

The original article has been corrected online to rectify this error.
